# A new species of the isopod genus *Ancinus* (Isopoda: Sphaeromatidae) from sandy beaches of the northern Yucatan Peninsula, Mexico

**DOI:** 10.1371/journal.pone.0321489

**Published:** 2025-04-30

**Authors:** Manuel Ortiz, María Teresa Herrera-Dorantes, Pedro-Luis Ardisson

**Affiliations:** 1 Laboratorio de Crustáceos, Facultad de Estudios Superiores Iztacala, Universidad Nacional Autónoma de México, Tlalnepantla, Estado de México, México; 2 Departamento de Recursos del Mar, Cinvestav Mérida, Mérida, Yucatán, México; The American College, INDIA

## Abstract

A new species of the sphaeromatid isopod genus *Ancinus* is described and illustrated. It comes from the Cinvestav Merida research project, Invertebrate Benthic Diversity of the Yucatan Intertidal and Shallow Subtidal Zones. This genus is easily recognized by having an oval and flattened body; in males, the first two pereonal appendages are subchelate, while in females, only the first one is subchelate, the pleotelson is triangular, and the first pleopods and uropods are uniramous. This new species of *Ancinus* differs from all others by body surface smooth and unpigmented; eyes not elevated; suture between head and pereonite 1 complete, straight; mandible lacinia mobilis lateral margins parallel; 3 irregular teeth, 1 long, 2 short blunt sclerotized cusps; mandible palp third article inner margin with 12 blunt robust distal setae; 2 maxilliped coupling setae; coxae 5–7 visible in dorsal view; pleopod 2 endopod margin with 20 short setae; male appendix styliform, fluted, and shorter than endopod; curved tip. This is the third known species of *Ancinus* in the Gulf of Mexico and the tenth worldwide. A table with the main differences among the three known species in the gulf is also given.

## Introduction

The genus *Ancinus* (Crustacea, Isopoda) groups sphaeromatid isopods distributed in the tropical and subtropical ecosystems of the western Atlantic and eastern Pacific. It includes nine known species [1–5]. Among them, two species in the Gulf of Mexico (*A*. *depressus* (Say, 1818) and *A*. *jarocho* Rocha-Ramírez, Chávez-López & Peláez-Rodríguez, 2010), one in the Caribbean Sea (*A*. *belizensis* Kensley & Schotte, 1987), and three in Brazilian waters (*A*. *braziliensis* Lemos de Castro, 1959, *A*. *gaucho* Pires, 1987 and *A*. *velella* Guedes-Silva, Paiva, Maida & Souza-Filho, 2019). The remaining three species, *A*. *granulatus* Holmes & Gay, 1909, *A*. *panamensis* Glynn & Glynn, 1974, and *A*. *seticomvus* Trask, 1971, are primarily reported from the eastern Pacific Ocean.

Until the work of Rocha-Ramirez et al. (2010), specimens of the genus had not been explicitly reported on sandy beaches of the Gulf of Mexico. Based on the available evidence, their presence in this ecosystem seems discrete, focused on specific regions, and not widely distributed. *A*. *depressus* has been reported from Georgia to Texas in the United States, and from Tamaulipas to Veracruz in Mexico, *A*. *jarocho* only from Veracruz (Mexico), and now *A. yucatanensis* **sp**. **nov**. from Yucatan (Mexico).

The genus *Ancinus* faces synonymy issues, as is common in many taxonomic groups, particularly those with subtle morphological differences and limited molecular data [1,3,6,7]. These issues arise due to challenges in distinguishing species based on morphology alone. While some references do not explicitly address synonymy, they provide descriptions of *Ancinus* species and highlight the morphological similarities that could lead to taxonomic confusion [2,4,8]. Historical descriptions with insufficient detail and the discovery of cryptic species (morphologically similar but genetically distinct) could contribute to worsening the problem. Although the existence of cryptic species has not been documented within the genus *Ancinus*, it has been documented in other genera of isopods, particularly from deep-sea and Antarctic environments [9–11].

This genus consists primarily of free-living, benthic isopods. These isopods are typically found in marine environments, such as intertidal and shallow tidal zones, where they live among algae, rocks, or sediment [6,7,12–17]. Nevertheless, some studies have signaled two isopods behaving like parasitic species, a significant and surprising finding on the life strategies of this genus that deserves attention; among these can be mentioned those of Villalba-Vasquez et al. [18] and Miranda‐Delgado et al. [19] reporting, respectively, to *Ancinus depressus* in the gills of the fish species *Parapsettus panamensis* and *Euthynnus lineatus* on the Mexico Pacific coast, and Nogueira Jr and Silva [20] reporting to *A*. *brasiliensis* in the subumbrella and external wall of the manubrium of the cubozoan *Chiropsalmus quadrumanus* on the littoral of Parana e Santa Catarina, Brazil.

These isopods are characterized by oval and flattened bodies, not more than 7–8 mm long. Males have their first two appendages subchelated, while females only have the first one subchelated. Its pleotelson is triangular, and first pleopods and uropods have single ramus.

This paper describes and illustrates a new species of sphaeromatid isopod of the genus *Ancinus*. This species is the third known in the Gulf of Mexico and the tenth worldwide. We also present a diagnostic table describing this area’s two known *Ancinus* species.

## Materials and methods

**Fig 1:**
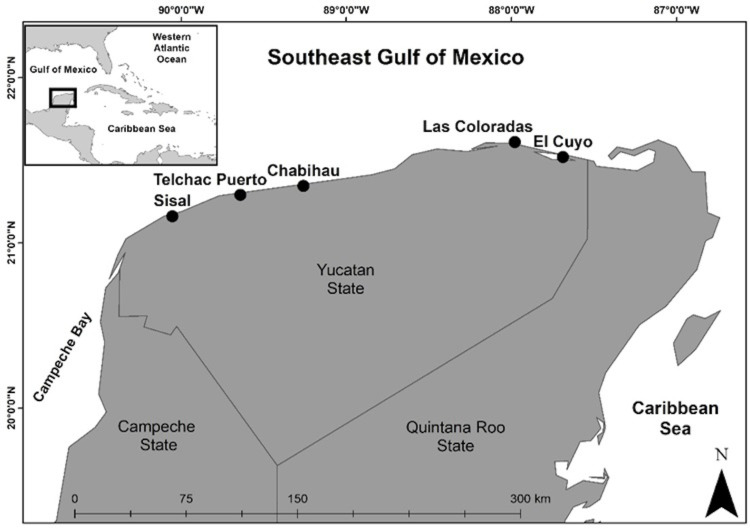
Study area. *Ancinus* collecting localities in the Yucatan Peninsula, Mexico.

The *Ancinus* specimens were hand-collected in 2011 on sandy carbonate-rich beaches of the northern Yucatan coast, southeast Gulf of Mexico (Fig 1), as part of the Cinvestav Merida research project, Invertebrate Benthic Diversity of the Yucatan Intertidal and Shallow Subtidal Zones. The specimens were collected on red algae in the swash zone. Male holotype and female and male paratypes were sorted under stereoscope microscopes. Photographs of male and female dorsal views were taken with an AxioCamERc5s camera coupled to a compound microscope (Primo Star Zeiss). Figures were inked with the program Corel Draw X-6. The type material is deposited in the Colección Nacional de Crustáceos (CNCR), Instituto de Biología, UNAM, Mexico.

### Nomenclatural acts

The electronic edition of this article conforms to the requirements of the amended International Code of Zoological Nomenclature, and hence the new names contained herein are available under that Code from the electronic edition of this article. This published work and the nomenclatural acts it contains have been registered in ZooBank, the online registration system for the ICZN. The ZooBank LSIDs (Life Science Identifiers) can be resolved and the associated information viewed through any standard web browser by appending the LSID to the prefix “http://zoobank.org/”. The LSID for this publication is: urn:lsid:zoobank.org:pub:11FA12DB-7093-41AF-82BC-0E40FCC8F1BC. The electronic edition of this work was published in a journal with an ISSN, has been archived, and is available from the following digital repositories: PubMed Central and LOCKSS.

## Results

### Taxonomy

Order Isopoda Latreille, 1825
Suborder Sphaeromatidea Wägelle, 1989
Superfamily Sphaeromatoidea Latreille, 1825
Family Ancinidae Dana, 1852
Genus *Ancinus* H. Milne Edwards, 1840

#### *Ancinus yucatanensis* sp. nov.

**Fig 2:**
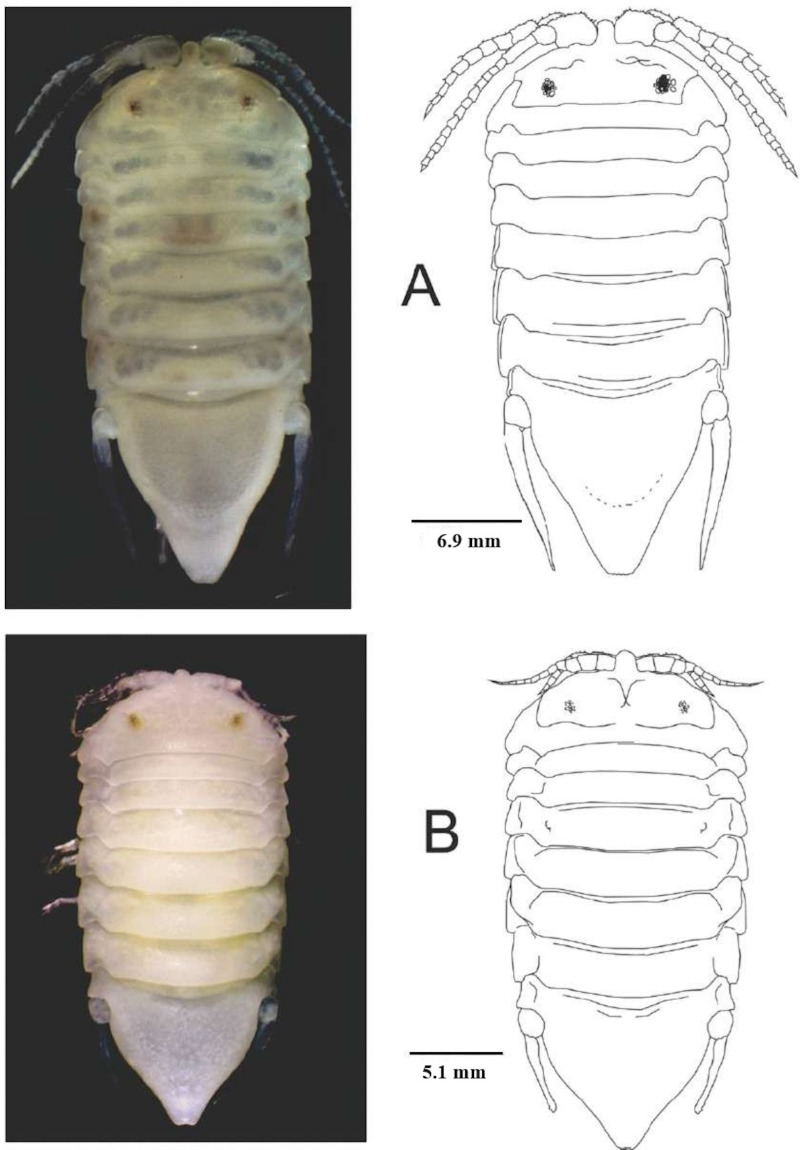
*Ancinus yucatanensis* sp. nov. A, dorsal views of male; B, same of female.

**Fig 3:**
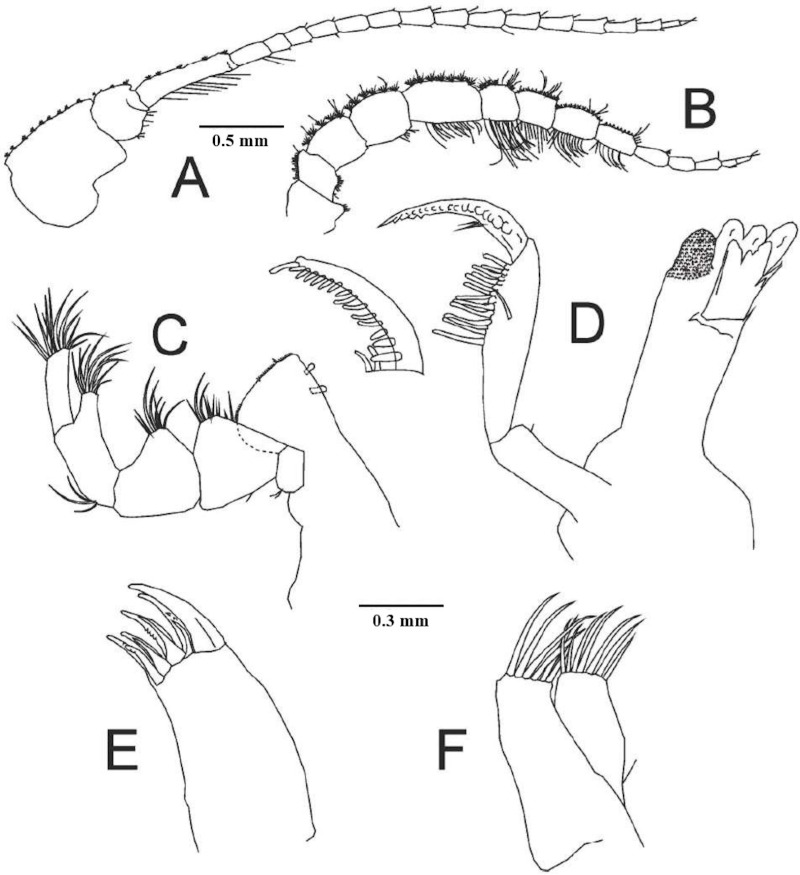
*Ancinus yucatanensis* sp. nov. A, antennula; B, antenna; C, maxilliped; D, mandible, E, maxilla 1; F, maxilla 2.

**Fig 4:**
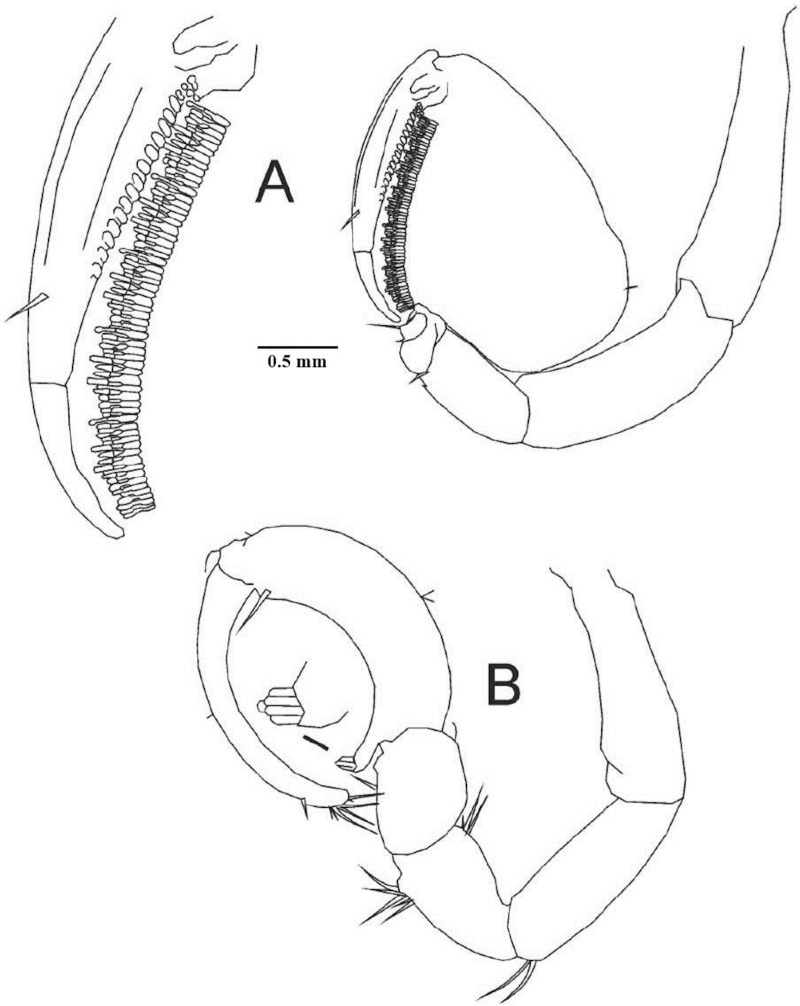
*Ancinus yucatanensis* sp. nov. A, pereopod 1; B, pereopod 2.

**Fig 5:**
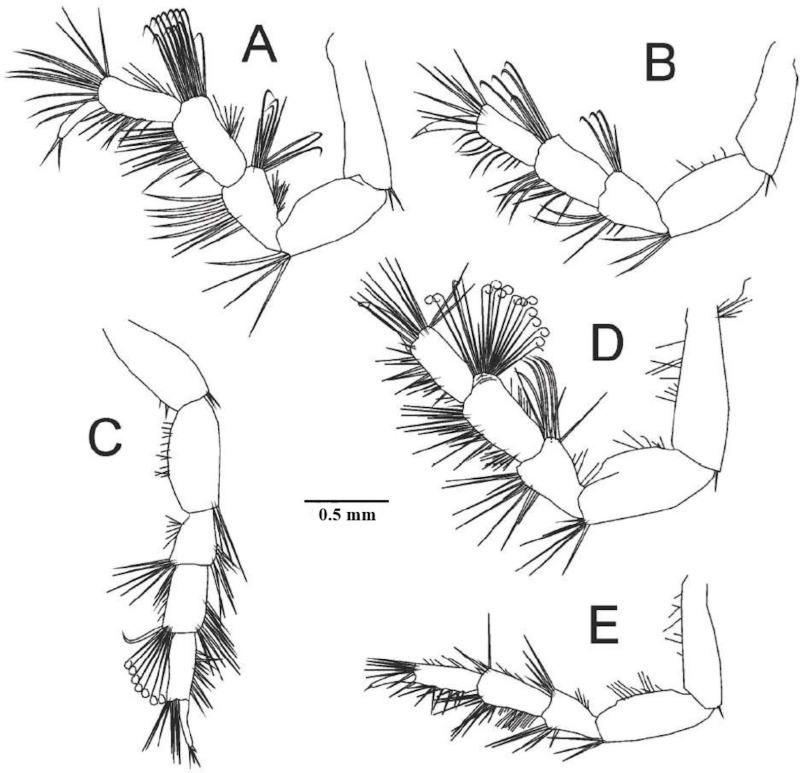
*Ancinus yucatanensis* sp. nov. A, pereopod 3; B, pereopod 4; C, pereopod 5; D, pereopod 6; E, pereopod 7.

**Fig 6:**
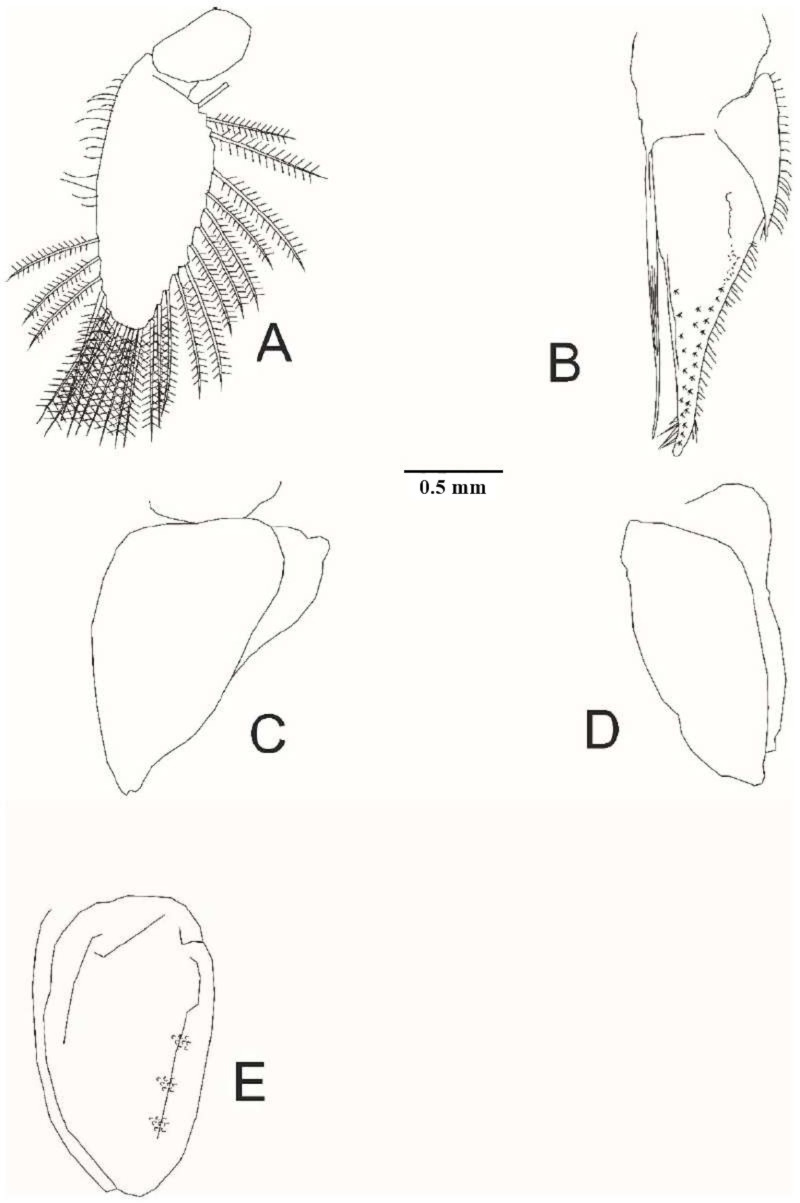
*Ancinus yucatanensis* sp. nov. A, pleopod 1; B, pleopod 2; C, pleopod 3; D, pleopod 4, E, pleopod 5.

urn:lsid:zoobank.org:act:C7F5DB19-9C36-4B62-AD1B-9472E450D5E0 (Figs 2–6).

### Type material

**Holotype.** Adult male, 6.97 mm total length (CNCR 37510), collected on 19 February 2011; Mexico, Yucatan State, Sisal (21°9’39.98“N, 90°3’13.21”W), Municipio de Hunucmá, María Teresa Herrera-Dorantes, collector.

**Paratypes.** Thirty-six specimens in total. Sisal (21°9’39.98“N, 90°3’13.21”W) (CNCR 37511, CNCR 37512, CNCR 37513, CNCR 37514, CNCR 37515, CNCR 37516); Telchac Puerto (21°17’24.7”N, 89°38’30.6”W) (CNCR 37517); Chabihau (21°20’39.2”N, 89°15’35”W) (CNCR 37518); Las Coloradas (21°36’34”N, 87°59.4’59W) (CNCR 37519, CNCR 37520, CNCR 37521, CNCR 37522, CNCR 37523, CNCR 37524, CNCR 37525, CNCR 37526, CNCR 37527, CNCR 37528, CNCR 37529, CNCR 37530, CNCR 37531, CNCR 37532, CNCR 37533, CNCR 37534, CNCR 37535, CNCR 37536, CNCR 37537); El Cuyo (21°31’9.24”N, 87°41’19”W) (CNCR 37538, CNCR 37539, CNCR 37540, CNCR 37541, CNCR 37542, CNCR 37543, CNCR 37544, CNCR 37545, CNCR 37546); same date and collector as holotype.

**Diagnosis.** Freshly collected, unpigmented body; 2.5 times as long as wide; pereon segment 1 widest; rostrum rounded apically; 0.7 as long as basal width of the antennule first article; eyes small; rounded; not elevated on swellings; first pereonite 0.8 as long as pereonite 2; pereonites 2 and 3 of the same length; pereonites 5–7, the longest; coxae 5–7 visible in dorsal view; maxilliped; palp articles 1 and 2 elongate with 18 setae; articles 3 and 4 with inner lobed centered; mandibles three articulate palps; distal article 14 robust blunt setae; left mandible incisors one long and two short blunt sclerotized cusps; one shallow none-sclerotized cusp; lacinia mobilis lateral margin parallel; not narrow basally; pereopod 1 propodus basal lobe not prominent; pereopod 2 dactylus exceeds palmar angle; basal posterior lobe 5 parallel outward directed short setae; uropod as long as the telson; telson tip truncate.

**Male holotype.** Body 2.5 longer than wide; surface smooth; devoid of stains; head completely embedded in pereonite 1; rostrum rounded; flagellar articles on both antennae without aesthetascs; eyes small rounded not elevated; interocular space 0.4 times head width; first pereonite 0.8 broadest; pereonites 2 and 3 of the same length; 5–7 the longest; coxae 5–7 visible in dorsal view; uropod slightly curved inward; telson triangular and truncate 1.2 longer than wide; smooth (Fig 2).

**Antennule** article 1 as long as 3; second article the shortest; 16 articles flagellum (Fig 3A).

Antenna article 5 longer than 4; 8 articles flagellum (Fig 3B). **Maxilliped** 2 robust setae inner margin; palp articles 2–4 inner distal margin produced into lobes; lobe article 3 centered; lobe article 2 with 18 and article 4 with 11 curved robust setae; half lobe of article 4 in article 5, article 5 with 13–15 setae on the distal margin (Fig 3C). **Mandibles** stout; left mandible incisors 1 long, 2 short blunt sclerotized cusps; 1 shallow none-sclerotized cusp; lacinia mobilis wide not narrowing basally; palps article 1 the shortest; article 2 the longest; 1.2 as long as 3; distal half inner margin 12 curved setae; article 3 with 14 robust blunt setae (Fig 3D). **Maxilla 1** outer plate discreetly converging towards distal margin; 8 robust distal setae arranged in 2 rows; outer setae big; 3 inner row setae; 4 outer setae. Inner plate reduced (3E). **Maxilla 2** outer plate higher than inner; 6 long distal setae; 7 long inner plate distal setae (3F). **Pereopod 1** basis narrow basally; 1.4 as long as ischium; merus rectangular; 2 times longer than carpus; carpus short; quadrangular; propodus very inflated on its back; propodus basal lobe not prominent; quadrangular; parallel truncated distal widened setae palmar margin; dactylus fixing palm; pectinate basal half inner margin; 1 seta outer margin; unguis short; as length as ischium width (Fig 4A). **Pereopod 2** basis tapering distally; long as ischium; 2 setae posterodistal angle; merus 0.2 longer than carpus; tuft of 7 setae posteroventral margin; 2 anterodistal margin setae; carpus 1 setae on the anterior and posterior margins; propodus strongly curved; anterior margin concave; of same thickness throughout its entire length; basal posterior lobe 5 parallel outward short setae; single seta distal posterior angle; dactylus exceeds palmar angle; unguis flat (Fig 4B). **Pereopod 3** ischium wider than basis; anterodistal long tip curved setae; posterodistal long setae; propodus narrower than carpus; dactylus 0.7 as long as carpus; unguis absent (Fig 5A). **Pereopod 4** basis as long as ischium; merus-carpus anterodistal tuft curved tip setae; dactylus as long as propodus; unguis present; short (Fig 5B). **Pereopod 5** basis as long as ischium; merus antero and posterodistal setae present; carpus anterodistal tuft of circle tip setae; dactylus unguis present, short (Fig 5C).

**Pereopod 6** broad carpus tuft of anterodistal tip circle setae; propodus shorter and narrower than carpus; long set distal setae; dactylus as long as propodus; unguis present (Fig 5D).

**Pereopod 7** ischium as long as basis; merus postero and antero tuft distal setae; carpus posterior margin 2 tuft of long setae; propodus anterodistal tuft of setae; dactylus. 0.7 as long as propodus; unguis present (5E). **Pleopod 1** bearing 25 long plumose setae along margins (Fig 6A). **Pleopod 2** exopod triangular; pointed distal corner; 20 lateral short setae; endopod 2 times longer than protopodite; pointed distally; short setae outer margin; male appendix styliform; fluted shorter than endopod; curved tip (Fig 6B). **Pleopod 3** triangular; without branched (Fig 6C). **Pleopod 4** rectangular; without branched (Fig 6D). **Pleopod 5** ovoidal; exopod as long as endopod; 3 squamiferous protuberances (Fig 6E).

**Female**. Similar to male; smaller; rostrum 1.2 as long as basal width of antennule first article; suture between head and pereonite 1 incomplete; sinuous; telson 1.2 wider than long; uropods shorter than the telson.

**Etymology.** “yucatanensis” honors the type locality in the Yucatan Peninsula, Mexico.

## Discussion

A recent paper provided an excellent table comparing selected morphological characters and a dichotomic key to separate the nine *Ancinus* species known until now [4]. Therefore, only the most important characteristics that separate the three known species in the Gulf of Mexico (*Ancinus depressus, A. jarocho,* and *A. yucatanensis* **sp**. **nov**.) are presented below (Table 1).

**Table 1:** Main morphologic characteristics to separate the three species of *Ancinus* recorded in the Gulf of Mexico up to date.

Characteristic	Species		
	*Ancinus depressus*	*Ancinus jarocho*	*Ancinus* *yucatanensis* **sp. nov.**
**Maximum body width**	pereonite 5	pereonites 2–4	pereonite 1
**Body surface**	smooth, pigmented?	granulose, pigmented	smooth, unpigmented
**Eyes elevated**	not	yes	not
**Suture between head and pereonite 1**	incomplete, curved	incomplete, curved	complete, straight
**Mandible lacinia mobilis**	wide, narrowing basally, 2 long and 2 short teeth	narrow, 2 sclerotized and 2 non- sclerotized cusps	wide, lateral margins parallel, 3 irregular teeth, 1 long, 2 short blunt sclerotized cusps
**Mandible palp third article**	tuft of short distal setae; half the length article 2	10 pointed setae in distal half, 0.6 as long as second	Inner margin covered with 12 blunt robust distal setae
**Maxilliped palp inner lobe article three**	centered	distal	centered
**Maxilliped coupling setae**	1	1	2
**Coxae 5–7**	visible in dorsal view	not visible in dorsal view	Visible in dorsal view
**Pereopod 1 propodus and proximal process**	Rounded, short, not prominent	Rectangular, prominent	very short, curved, not prominent
**Pereopod 2 dactylus**	?	short, curved	elongate, curved
**Pereopod 2 propodus and proximal process**	?	4 robust setae, dactylus tip reaching base of propodus	5 blunt, simple short setae, tip surpasses the base of propodus
**Pereopod 3, 5 & 7 carpus anterior lobe**	?	tuft of simple setae, pigmented	tuft of curved tip setae
**Pleopod 2 endopod margin**	?	sparse plumose with 24 long simple setae	complete covered with 20 short setae
**Pleopod 5 squamigerous prominences**	5	3	3

After this table, the three species share several common characteristics. Therefore, the most appropriate way to differentiate the new species from the other two is by referring to the maximum body width (at the level of the pereonite 1 in the new species and the pereonites 2–4 in the others). For greater classification accuracy, it is possible to observe the type of mandible lacinia mobilis, which is wide and has parallel margins in *A. yucatanensis* **sp**. **nov**. instead of narrow in *A*. *jarocho*, and lateral margins converging towards the base in *A*. *depressus*; body surface smooth and unpigmented in *A. yucatanensis* **sp**. **nov**. instead of granulose and pigmented in the rest; mandible palp inner margin covered with 15 blunt robust distal setae in *A. yucatanensis* **sp**. **nov**. instead of less than 10; pereopod 2 dactylus surpassing the palm in *A. yucatanensis* **sp**. **nov**. instead of dactylus fixing palm; pereopod 3, 5 & 7 carpus anterior lobe tuft of circle tip setae in *A. yucatanensis* **sp**. **nov**. instead of curved or simple setae in *A. depressus* and *A. jarocho*.
